# Anacardic Acids from Cashew Nuts Ameliorate Lung Damage Induced by *Exposure* to Diesel Exhaust Particles in Mice

**DOI:** 10.1155/2013/549879

**Published:** 2013-02-27

**Authors:** Ana Laura Nicoletti Carvalho, Raquel Annoni, Larissa Helena Lobo Torres, Ana Carolina Cardoso Santos Durão, Ana Lucia Borges Shimada, Francine Maria Almeida, Cristina Bichels Hebeda, Fernanda Degobbi Tenorio Quirino Santos Lopes, Marisa Dolhnikoff, Milton Arruda Martins, Luiz Fernando Ferraz Silva, Sandra Helena Poliselli Farsky, Paulo Hilário Nascimento Saldiva, Cornelia M. Ulrich, Robert W. Owen, Tania Marcourakis, Maria Teresa Salles Trevisan, Thais Mauad

**Affiliations:** ^1^Experimental Atmospheric Pollution Laboratory (LPAE), Department of Pathology, São Paulo Medical School, University of São Paulo, Av. Dr. Arnaldo 455/Room 1155, 01246-903 São Paulo, SP, Brazil; ^2^Department of Clinical and Toxicological Analyses, School of Pharmaceutical Sciences, University of São Paulo, Av. Prof. Lineu Prestes 580/13b, 05508-900 São Paulo, SP, Brazil; ^3^Experimental Therapeutics Laboratory, Department of Clinical Medicine, São Paulo Medical School, University of São Paulo, Av. Dr. Arnaldo 455/Room 1155, 01246-903 São Paulo, SP, Brazil; ^4^National Institute for Integrated Analysis of Environmental Risk (INAIRA), National Council for Scientific and Technological Development, Av. Dr. Arnaldo 455/Room 1220, 01246-903 São Paulo, SP, Brazil; ^5^Division of Preventive Oncology, National Center for Tumor Diseases, Im Neuenheimer Feld 460/German Cancer Research Center (DKFZ), Im Neuenheimer Feld 581, Heidelberg, Germany; ^6^Department of Organic and Inorganic Chemistry, Federal University of Ceará, Campus 12200, 60451-970 Fortaleza, CE, Brazil

## Abstract

Anacardic acids from cashew nut shell liquid, a Brazilian natural substance, have antimicrobial and antioxidant activities and modulate immune responses and angiogenesis. As inflammatory lung diseases have been correlated to environmental pollutants exposure and no reports addressing the effects of dietary supplementation with anacardic acids on lung inflammation *in vivo* have been evidenced, we investigated the effects of supplementation with anacardic acids in a model of diesel exhaust particle- (DEP-) induced lung inflammation. BALB/c mice received an intranasal instillation of 50 **μ**g of DEP for 20 days. Ten days prior to DEP instillation, animals were pretreated orally with 50, 150, or 250 mg/kg of anacardic acids or vehicle (100 **μ**L of cashew nut oil) for 30 days. The biomarkers of inflammatory and antioxidant responses in the alveolar parenchyma, bronchoalveolar lavage fluid (BALF), and pulmonary vessels were investigated. All doses of anacardic acids ameliorated antioxidant enzyme activities and decreased vascular adhesion molecule in vessels. Animals that received 50 mg/kg of anacardic acids showed decreased levels of neutrophils and tumor necrosis factor in the lungs and BALF, respectively. In summary, we demonstrated that AAs supplementation has a potential protective role on oxidative and inflammatory mechanisms in the lungs.

## 1. Introduction

Epidemiological studies have clearly associated ambient particulate matter (PM) concentration with a range of adverse effects on respiratory and cardiovascular health as well as increased morbidity and mortality [1–3]. 

Diesel engine exhaustion contributes considerably to the air particulate composition in urban regions. In São Paulo, for instance, a fleet of 14,900 buses powered by diesel fuel are responsible for the majority of public transportation [4]. In healthy subjects, acute diesel exhaust exposure resulted in neutrophils recruitment, upregulation of the endothelial adhesion molecules P-selectin, and vascular adhesion molecule- (VCAM-1) and interleukin- (IL-8) production in the bronchial mucosa [2, 5, 6]. Subchronic exposure to lower levels of diesel exhaust particles (DEP) (30 *µ*g) derived from the São Paulo public transportation system has been shown to induce inflammatory alterations in the nose and lungs of healthy mice [7].

The mechanisms by which DEP induces adverse biologic effects on the respiratory system may be *via* the production of oxidative stress by the exposed cells [2, 8, 9]. Riedl and Diaz-Sanchez [10] observed that DEP exposure may cause increased oxidative stress directly through the induction of reactive oxygen species (ROS) and indirectly through the resultant enhanced inflammation, which generates additional ROS. Additionally, DEP activates redox-sensitive transcription factors, such as nuclear factor kappa B (NF-*κ*B) and activator protein- (AP-1) [2, 10, 11]. 

While there clearly need to be efforts worldwide to reduce diesel related air pollution, identifying potential protective substances that reduce the harmful respiratory effects of pollutant-induced oxidative stress has been an important research topic in recent years [2, 10]. Diet is the only source of antioxidant micronutrients [12], and these micronutrients are thought to be important modulators of immune response [2, 13–15]. Antioxidants can upregulate endogenous antioxidant defenses, such as reduced glutathione (GSH), catalase, and glutathione reductase [16, 17]. 

Due to their wide distribution in fruits and plants, polyphenols are the most abundant antioxidants in the diet. The beneficial health effects of dietary polyphenols have recently come to the attention of nutritionists [18]. 

Anacardic acids (AAs) are alkyl phenols from the cashew (*Anacardium occidentale* Linn.), a tropical tree native to the northeast region of Brazil. AAs are abundantly present in many parts of the cashew plant and have received attention as a potential antioxidant substance. Cashew apple, cashew nut (raw and roasted), and cashew nut shell liquid (CNSL) contain a range of different alkyl phenols, including AAs, cardanols, and cardols. Higher amounts of AAs have been detected in CNSL (353.6 g/kg) followed by cashew fiber (6.1 g/kg), while the lowest (0.65 g/kg) amounts were found in roasted cashew nut [19].

 AAs were described as the main active agent in CNSL. The presence of a phytyl side chain beside the phenolic ring structure (as in salicylic acid) results in its great antioxidant capacity [19]. Diverse biological activities for the AAs have been described, including antimicrobial activity against methicillin-resistant bacteria [20–22], gastroprotection [23], and inhibition of the activity of several clinically targeted enzymes, such as lipoxygenase [24, 25], cyclooxygenase [26, 27], and histone acetyltransferases [28, 29]. It has been also demonstrated that AAs modulate the NF-*κ*B signaling pathway and inhibit tumor angiogenesis indicating that these compounds could be a therapeutic option in preventing or treating cancer [30–32]. 

To date, very few *in vivo* studies have tested the effects of AAs. Morais et al. [23] suggested that AAs induce gastroprotection primarily through an antioxidant mechanism at 10, 30, and 100 mg/kg. We have previously demonstrated that doses of AAs less than 300 mg/kg do not produce biochemical, hematological, and mutagenic alterations in BALB/c mice [33]. 

We hypothesized that AAs from CNSL would prevent DEP-induced lung inflammation. Based on this hypothesis, we analyzed the potential anti-inflammatory and antioxidant properties of supplementation with AAs in a subacute model of DEP-induced inflammation in mice.

## 2. Materials and Methods

### 2.1. Ethics Statement

 This study was approved by the Ethical Committee of São Paulo University Medical School (permit number: 114/07). All animals received care in compliance with the “Principles of Laboratory Animal Care” published by the National Institutes of Health. All surgery procedure was performed under anesthesia, and all efforts were made to minimize suffering.

### 2.2. Animals

Six- to eight-week-old male BALB/c mice (20–25 g) were obtained from the animal facility of São Paulo Medical School, University of São Paulo. Animals were housed in group cages at 22–26°C with a 12 h/12 h light/dark cycle and received *ad libitum* water and commercial pellet food for small rodents from Nuvital (Nuvilab CR-1; Colombo, Brazil).

### 2.3. Plant Material

The cashews (*Anacardium occidentale *Linn.) were harvested at the Embrapa Tropical Agroindustry Experimental Station, located at Paraipaba, Ceará, Brazil, during the 2007 season. The fruits were from a commercial cultivar (CCP-76), whose genetic material is maintained on the Embrapa's germplasm bank. The fresh cashew apples were manually separated from the nut and were obtained as a kind gift from Dr. Edy Sousa de Brito (Embrapa, Fortaleza, Brazil). CNSL (300 g) was obtained by heating 1 kg of fruit (175°C) in an oven for 45 min. The cashew nuts oil (2 L) was obtained from cashew nuts subjected to Soxhlet extraction with hexane (3 h) [19].

### 2.4. Isolation of AAs from CNSL

**Figure 1:**
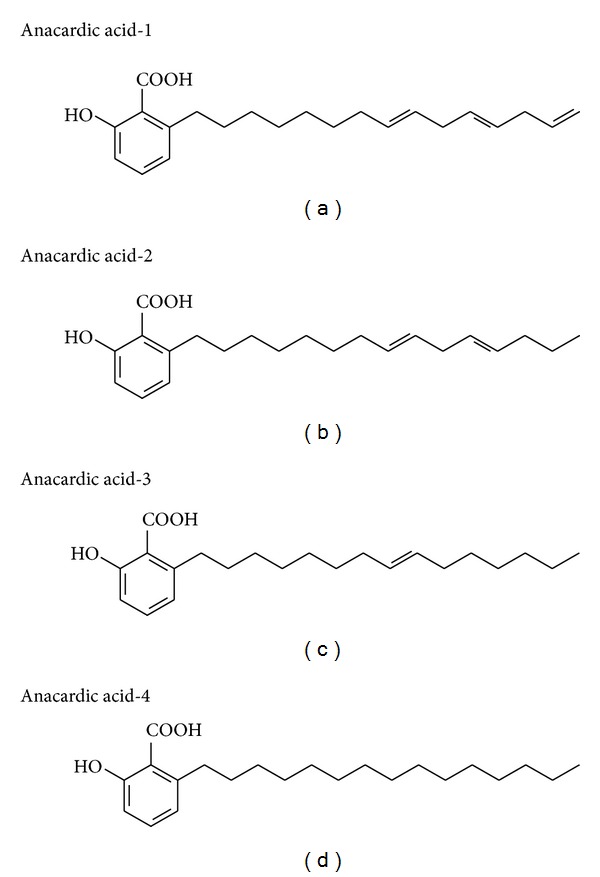
Chemical structure of anacardic acids.

Extracted CNSL (200 g) was dissolved in 5% aqueous methanol (1200 mL), and calcium hydroxide (100 g) was added while stirring. The mixture was kept at 50°C and stirred for 3 h. The supernatant solution was monitored by two-dimensional thin layer chromatography for the absence of AAs. The precipitated calcium anacardate was filtered and washed with methanol. Calcium anacardate was dissolved in distilled water acidified with 11 M HCl. The solution was extracted with ethyl acetate; the ethyl acetate layer was then washed with distilled water, dried over anhydrous sulfate, and concentrated under reduced pressure to yield 120 g of AAs mixture, as described by Paramashivappa et al. [34]. All structures were verified by comparing spectral and physical data with those previously reported in the literature [19] and by direct comparison with authentic samples.  Figure 1 depicts the structure of the AAs isolated from CNSL.

### 2.5. DEP Collection

Diesel particles were collected from a bus from São Paulo city's metropolitan fleet after one day of routine operation. This bus was equipped with a Mercedes Benz MB1620, 210-hp engine with a Euro III emission profile, which lacked an electronic fuel injector. The diesel fuel used in São Paulo contains 500 ppm of sulphur. The 6 to 7 *μ*m diesel particulate material was collected with a particle retainer (a bimetallic filter) that is used to test diesel vehicles to reduce PM emissions. Particulate material was stored at 4°C for toxicological and analytical studies. DEP was dissolved in saline at 10 mg/mL for 2 h through magnetic stirring and was sonicated for 30 min. Next, DEP was diluted to 50 *μ*g in 10 *μ*L of saline and stored at −20°C until further use. Characteristics of the DEP used in this study have previously been evaluated by energy-dispersive X-ray fluorescence spectrometry to determine the metal composition, as well as by atomic absorption spectrophotometry to assess polycyclic aromatic hydrocarbons [7, 35].

### 2.6. Exposure Protocol and Supplementation with AAs

Lung inflammation in male BALB/c mice was induced by intranasal instillation of 50 *μ*g of DEP diluted in 10 *μ*L of saline solution for 20 consecutive days. The control group received 10 *μ*L of saline solution during the same period. Ten days prior to the intranasal instillation procedure, animals were pretreated orally with 50, 150, or 250 mg/kg of AAs from CNSL diluted in 100 *μ*L of cashew nut oil (CNO) or 100 *μ*L of CNO for 30 days. The three doses were selected based on previous dose-dependent studies in related specimens [23, 33, 36–39]. Eighty mice were assigned to five groups: (a) control (Ctrl) animals received an intranasal instillation of 10 *μ*L saline solution and were treated orally with 100 *μ*L of CNO (vehicle); (b) diesel exhaust particles (DEP) animals received an intranasal instillation of 50 *μ*g DEP/10 *μ*L of saline and were treated orally with 100 *μ*L of CNO; (c) diesel exhaust particles + AAs 50 mg/kg (DA50) animals received an intranasal instillation of 50 *μ*g DEP/10 *μ*L of saline and were treated orally with 50 mg/kg of AAs; (d) diesel exhaust particles + AAs 150 mg/kg (DA150) animals received an intranasal instillation of 50 *μ*g DEP/10 *μ*L of saline and were treated orally with 150 mg/kg of AAs; and (e) diesel exhaust particles  + AAs 250 mg/kg (DA250) animals received an intranasal instillation of 50 *μ*g DEP/10 *μ*L of saline and were treated orally with 250 mg/kg of AAs. From each group, eight animals were used for bronchoalveolar lavage fluid (BALF) collection and histological and immunohistochemistry analysis of the lungs. The remaining eight animals were used for analyzing the activity of antioxidant enzymes in lung homogenates and in erythrocytes.

### 2.7. BALF Collection and Analysis

Twenty-four hours following the last DEP (or saline) intranasal instillation and supplementation with AAs (or CNO), animals were anesthetized by intramuscular injection of ketamine (50 mg/kg) and xylazine (40 mg/kg), tracheostomized, and cannulated for BALF collection. BALF samples (1 mL) were collected after washing the lungs with 1.5 mL of phosphate buffered saline (PBS). BALF samples were centrifuged at 850 rpm for 10 min at 5°C, the supernatant was stored at −70°C, and the cell pellet was resuspended in 300 *μ*L of PBS. Total cell counts were performed using the Neubauer hemocytometer cell count chamber (Neubauer Improved Chamber, Labor Optik, Friedrichsdorf, Germany). Differential cell count was performed by microscopic examination of BALF samples prepared on cytocentrifuge slides (Cytospin-2, Shandon Instruments, Sewickley, PA) stained with Diff Quik (Muto Kagaku Co., Tokyo, Japan). Three hundred cells were counted per slide. The levels of IL-1*β*, tumor necrosis factor alpha (TNF-*α*), IL-6, and IL-10 in the BALF supernatants were determined. Cytokine concentrations were quantified using an enzyme-linked immunosorbent assay according to the manufacturer's protocol. The kit for IL-1*β* detection was obtained from eBioscience (San Diego, CA, USA), and the kits for TNF-*α*, IL-6 and IL-10 detection were purchased from BD Biosciences (Franklin Lakes, NJ, USA). Following BALF collection, the animals were sacrificed by exsanguinations, and the lungs were removed for histological and immunohistochemistry analysis.

### 2.8. Antioxidants Enzymes Determinations

Twenty-four hours following the final intranasal instillation of DEP (or saline) and supplementation with AAs (or CNO), animals were anesthetized, and the peripheral blood was collected. Then, the lungs were removed after perfusion with saline solution and were stored at −80°C. Enzymatic activities of glutathione peroxidase (GPx), glutathione reductase (GR), glutathione S-transferase (GST), and catalase (CAT) were determined in the lung homogenates and in erythrocytes using a spectrophotometric method. GPx activity was assessed with the procedure described by Flohé and Günzler [40]. Tert-butyl hydroperoxide was used as substrate, and the formation of oxidized glutathione (GSSG) was indirectly monitored spectrophotometrically as NAPDH consumption at 340 nm for 5 min. GR activity was assayed according to Carlberg and Mannervik [41]. The reduction of GSSG to GSH was measured as the consumption of NADPH and monitored spectrophotometrically at 37°C for 10 min at 340 nm. The GST activity assay, which measured the conjugation of 1-chloro-2,4-dinitrobenzene (CDNB) with reduced glutathione, was conducted according to Habig et al. [42]. The formation of the complex was monitored spectrophotometrically at 25°C for 5 min at 340 nm. GPx, GR, and GST assays were performed in a PowerWave ×340 Spectrophotometer (Bio-Tek Instruments INC, software KC4 v3.0). Catalase activity was evaluated by measuring the consumption of hydrogen peroxide [43]. The decrease in absorbance was monitored at 25°C for 30 s at 240 nm in a spectrophotometer (Biochrom Libra S12). 

### 2.9. Lung Histology, Morphometry and Immunohistochemistry

 Lungs were fixed in formalin and embedded in paraffin. Five-micrometer sections were stained with hematoxylin and eosin for the quantification of neutrophil density. 

#### 2.9.1. Morphometry

For conventional morphometry, an eyepiece with a coherent system of 50 lines and 100 points with a known area attached to the ocular lens of the microscope was used. The density of neutrophils in the alveolar parenchyma was assessed by point counting. Using a 100-point grid with a known area (7,000 *μ*m^2^ at 1000x magnification) attached to the microscope ocular lens, we counted the number of points hitting alveolar tissue in each field. Alveolar tissue area in each field was calculated as the number of points hitting alveolar tissue as a proportion of the total grid area. Neutrophil density was determined as the number of neutrophils in each field divided by the tissue area. Measurements are expressed as cells/mm^2^. Counting was performed in 40 fields of alveolar parenchyma for each animal at a magnification of 1000x [44].

#### 2.9.2. Immunohistochemistry

Lung sections were deparaffinized and hydrated. After blocking for endogenous peroxidase, antigen retrieval was performed with either high-temperature citrate buffer (pH = 6.0), tris-EDTA or trypsin. The following primary antibodies were used in the study: goat 8-epi-PGF_2*α*_ (8-isoprostane antibody) (goat, 1 : 500, Oxford Biomedical Research, Oxford, England), mouse Mac-2 (macrophage antibody) (mouse, 1 : 100,000, Cedarlane, ON, Canada), goat TNF-*α* (goat, 1 : 2,000, Santa Cruz Biotechnology, CA, USA), rabbit VCAM-1 (rabbit, 1 : 600, Santa Cruz Biotechnology, CA, USA), rabbit NF-*κ*B p65 (rabbit, 1 : 200, Santa Cruz Biotechnology, CA, USA), and mouse CXCL1/KC (keratinocyte chemoattractant (KC) antibody) (mouse, 1 : 100, Cedarlane, MN, USA). The VECTASTAIN ABC Kit, Vector Laboratories (Burlingame, CA, USA) was used as the secondary antibody, and 3,3-Diaminobenzidine (DAB, Sigma, St Louis, Mo, USA), was used as the chromogen. Tissue sections were counterstained with Harris hematoxylin (Merck, Darmstadt, Germany). For negative controls, the primary antibody was omitted from the procedure, and bovine serum albumin was used instead.

#### 2.9.3. Image Analysis

Areas positively immunostained for 8-isoprostane, macrophage cell density, and cells expressing KC, TNF-*α*, and NF-*κ*B in the alveolar parenchyma, as well as the areas positively immunostained for VCAM and KC in peribronchiolar vessels, were determined using image analysis. Analyses were performed using Image-Pro Plus 4.1 software for Windows (Media Cybernetics, Silver Spring, Md) on a personal computer connected to a digital camera (Olympus Q-Color 5, Tokyo, Japan) coupled to a light microscope. We counted the number of positively stained cells in the alveolar tissue in each field. The 8-isoprostane-stained regions and the alveolar tissue areas were calculated for 20 fields (alveolar tissue) at a magnification of 200x. Data are expressed as cell density per alveolar tissue area (cells/*μ*m^2^) or macrophages cell density per alveolar area (cells/mm^2^) and 8-isoprostane immunostained area per alveolar tissue area (*μ*m^2^/*μ*m^2^). We also assessed VCAM and KC positive areas in five peribronchiolar vessels [45]. The results were expressed as immunostained area per perimeter of the outer muscular layer of the vessel (*μ*m^2^/*μ*m). Slides were coded for blind analysis. All measurements were performed by the same observer.

### 2.10. Statistical Analyses

Statistical analyses were performed with SPSS 15.0 software (SPSS, Inc., Chicago, IL, USA). Data are expressed as the means ± standard deviation (SD) or as medians (interquartile range), unless otherwise specified. Comparisons between Ctrl and DEP, as well as between DEP versus DA50, DEP versus DA150, and DEP versus DA250, were performed using unpaired Student's *t*-tests (parametric data) or Mann-Whitney tests (nonparametric data). A *P* value of <0.05 was considered significant.

## 3. Results

### 3.1. BALF

As shown in Table 1, significant differences were not detected in BALF cell types between the Ctrl and DEP groups; however, an increase was observed in the number of neutrophils in the DA250 group and of lymphocytes in DA150 group compared to the DEP-treated group (*P* < 0.05). In the BALF supernatant, however (Table 2), levels of TNF-*α* were 6-fold higher in DEP-treated mice than in the Ctrl mice (*P* < 0.001). Mice treated with 50 mg/kg of AAs demonstrated decreased TNF-*α* levels, compared with mice treated with DEP (*P* < 0.002). The increased levels of IL-1*β*  were observed in the DA150 and DA250 groups, compared with the DEP group (*P* < 0.05). Equivalent levels of IL-6 and IL-10 levels were detected in BALF across all of the groups that were examined.

### 3.2. Antioxidant Enzyme Activities

**Figure 2:**
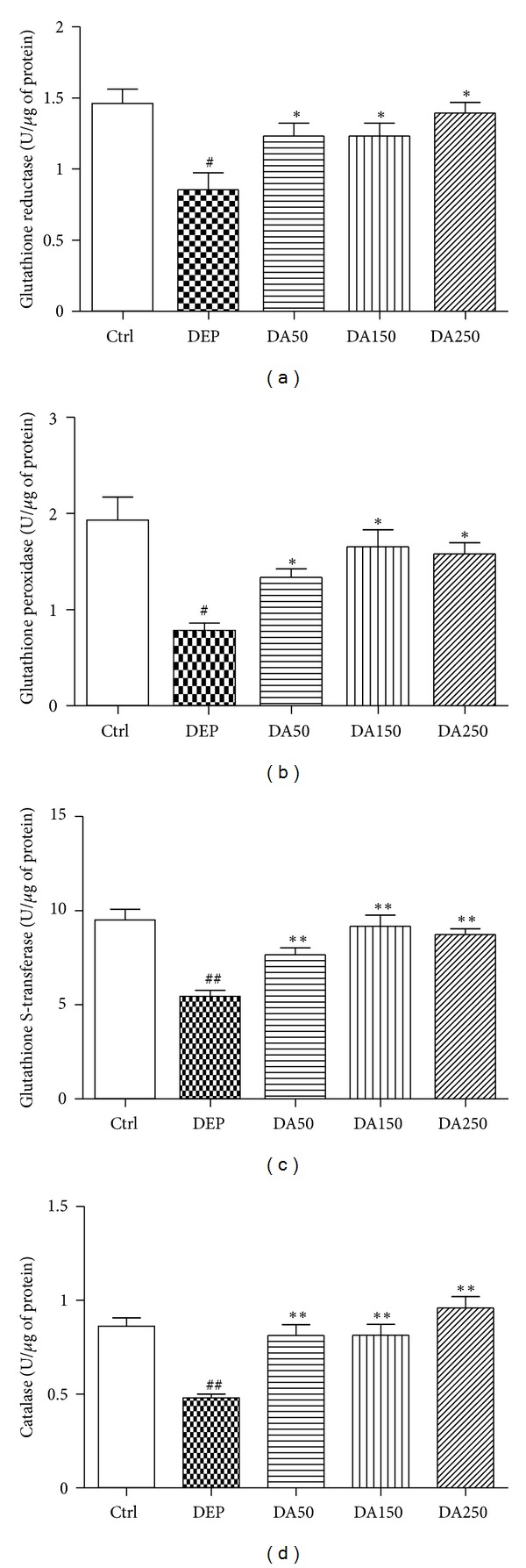
Glutathione reductase (a), glutathione peroxidase (b), glutathione S-transferase (c), and catalase (d) activities in lungs. Values represent means ± SEM. Ctrl: control; animals received an intranasal instillation of 10 *μ*L saline solution and were treated orally with 100 *μ*L of cashew nut oil (CNO). DEP: diesel exhaust particles; intranasal instillation of 50 *μ*g DEP/10 *μ*L of saline and treated orally with 100 *μ*L of CNO. DA50: intranasal instillation of 50 *μ*g DEP/10 *μ*L of saline and treated orally with 50 mg/kg of AAs. DA150: intranasal instillation of 50 *μ*g DEP/10 *μ*L of saline and treated orally with 150 mg/kg of AAs. DA250: intranasal instillation of 50 *μ*g DEP/10 *μ*L of saline and treated orally with 250 mg/kg of AAs. ^#^ *P* < 0.05 relative to Ctrl. *P < 0.05 relative to DEP. ^##^ *P* < 0.001 relative to Ctrl. **P < 0.001 relative to DEP.

**Figure 3:**
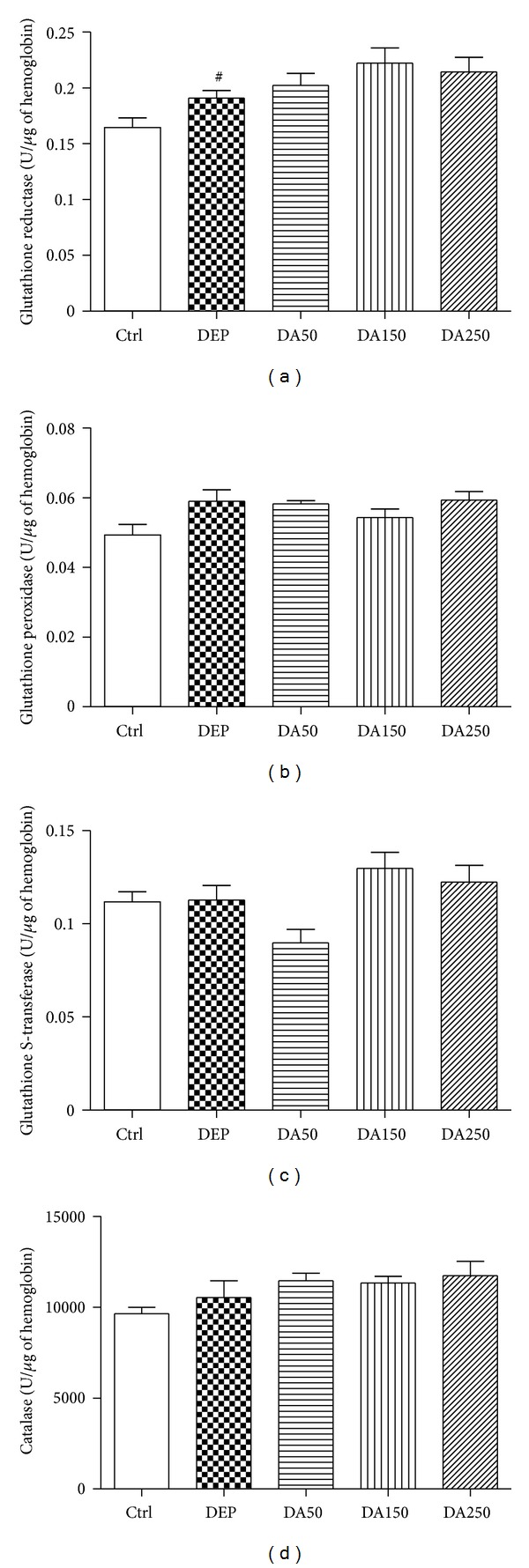
Glutathione reductase (a), glutathione peroxidase (b), glutathione S-transferase (c), and catalase (d) activities in blood. Values represent means ± SEM. Ctrl: control; animals received an intranasal instillation of 10 *μ*L saline solution and were treated orally with 100 *μ*L of cashew nut oil (CNO). DEP: diesel exhaust particles; intranasal instillation of 50 *μ*g DEP/10 *μ*L of saline and treated orally with 100 *μ*L of CNO. DA50: intranasal instillation of 50 *μ*g DEP/10 *μ*L of saline and treated orally with 50 mg/kg of AAs. DA150: intranasal instillation of 50 *μ*g DEP/10 *μ*L of saline and treated orally with 150 mg/kg of AAs. DA250: intranasal instillation of 50 *μ*g DEP/10 *μ*L of saline and treated orally with 250 mg/kg of AAs. ^#^ *P* < 0.05 relative to Ctrl.

Enzymatic analysis for GR (Figure 2(a)), GPx (Figure 2(b)), GST (Figure 2(c)), and CAT (Figure 2(d)) in lung homogenates all revealed the same pattern of activity. DEP-treated mice demonstrated decreased enzymatic activities, compared with the Ctrl mice (*P* < 0.05). Groups that received an oral supplementation with 50, 150, or 250 mg/kg of AAs showed significantly increased activity levels for all enzymes, compared with the DEP group (*P* < 0.05), especially for GST and CAT activities (*P* < 0.001). The DEP group showed increased activity of GR in the peripheral blood samples (Figure 3) relative to the Ctrl group (*P* < 0.05).

### 3.3. Inflammatory Response in Alveolar Parenchyma and in Peribronchiolar Vessels

**Figure 4:**
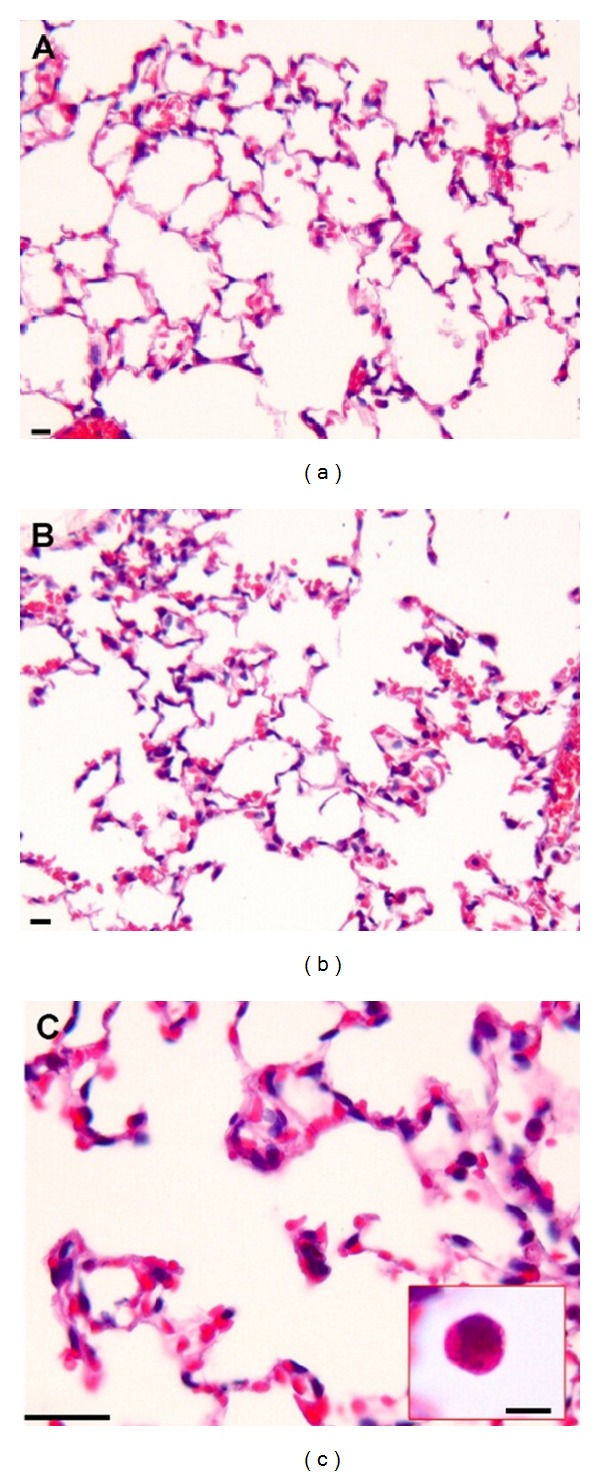
Photomicrographs showing a panoramic view of the inflammatory response in the lung tissue caused by DEP. The DEP group (b) demonstrated increased inflammatory response compared with the Ctrl group in the alveolar parenchyma (a). In detail (c), diesel particles phagocytized by a macrophage in the lung of an animal treated with DEP for 20 days. A black spot of anthracotic pigment can be observed in the inset (H&E). Scale bars = 25 *μ*m.

**Figure 5:**
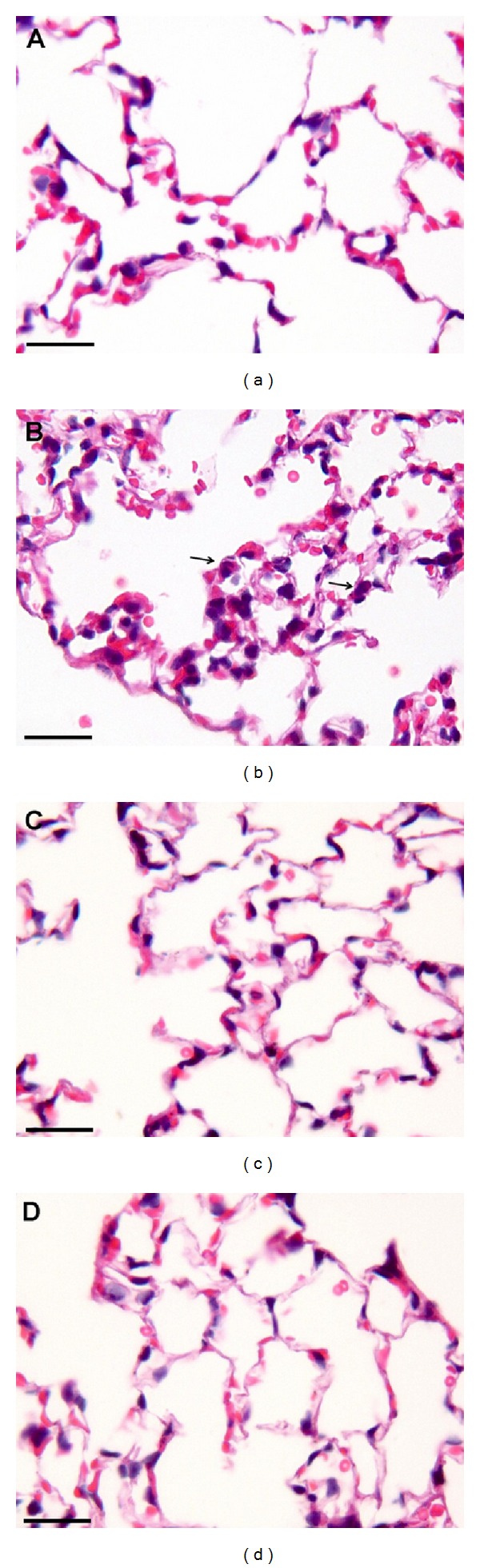
Photomicrographs showing the neutrophil density in lung tissue. The DEP group (b) demonstrated increased neutrophil density (in detail, the arrows are indicating some neutrophils) compared with the Ctrl group (a). Oral supplementation with 50 and 150 mg/kg of AAs was able to reduce the influx of neutrophils in lung tissue (c and d, resp.) (H&E). Scale bars = 25 *μ*m.

**Figure 6:**
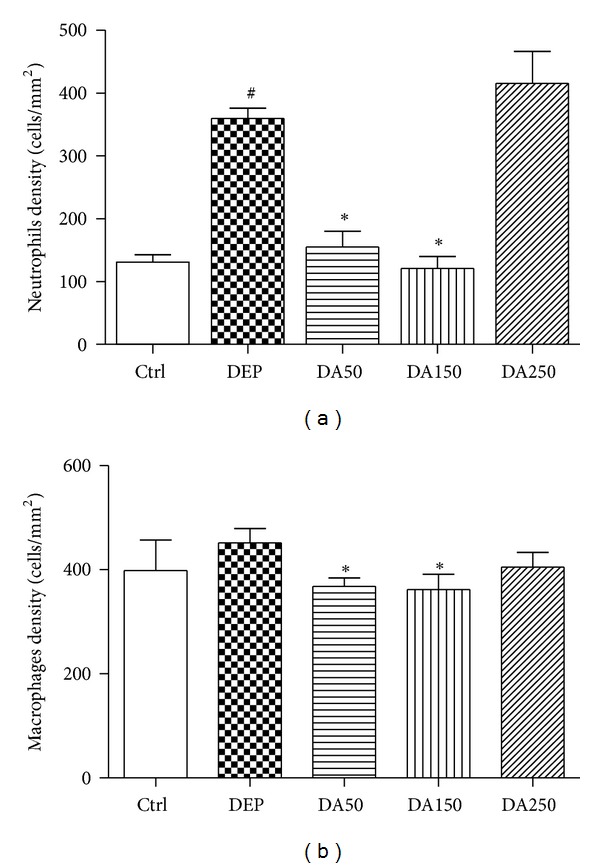
Influx of neutrophils (a) and macrophages (b) in the alveolar parenchyma. Values represent means ± SEM. Ctrl: control; animals received an intranasal instillation of 10 *μ*L saline solution and were treated orally with 100 *μ*L of cashew nut oil (CNO). DEP: intranasal instillation of 50 *μ*g DEP/10 *μ*L of saline and treated orally with 100 *μ*L of CNO. DA50: intranasal instillation of 50 *μ*g DEP/10 *μ*L of saline and treated orally with 50 mg/kg of AAs. DA150: intranasal instillation of 50 *μ*g DEP/10 *μ*L of saline and treated orally with 150 mg/kg of AAs. DA250: intranasal instillation of 50 *μ*g DEP/10 *μ*L of saline and treated orally with 250 mg/kg of AAs. ^#^ *P* < 0.001 relative to Ctrl. *P < 0.05 relative to DEP.

**Figure 7:**
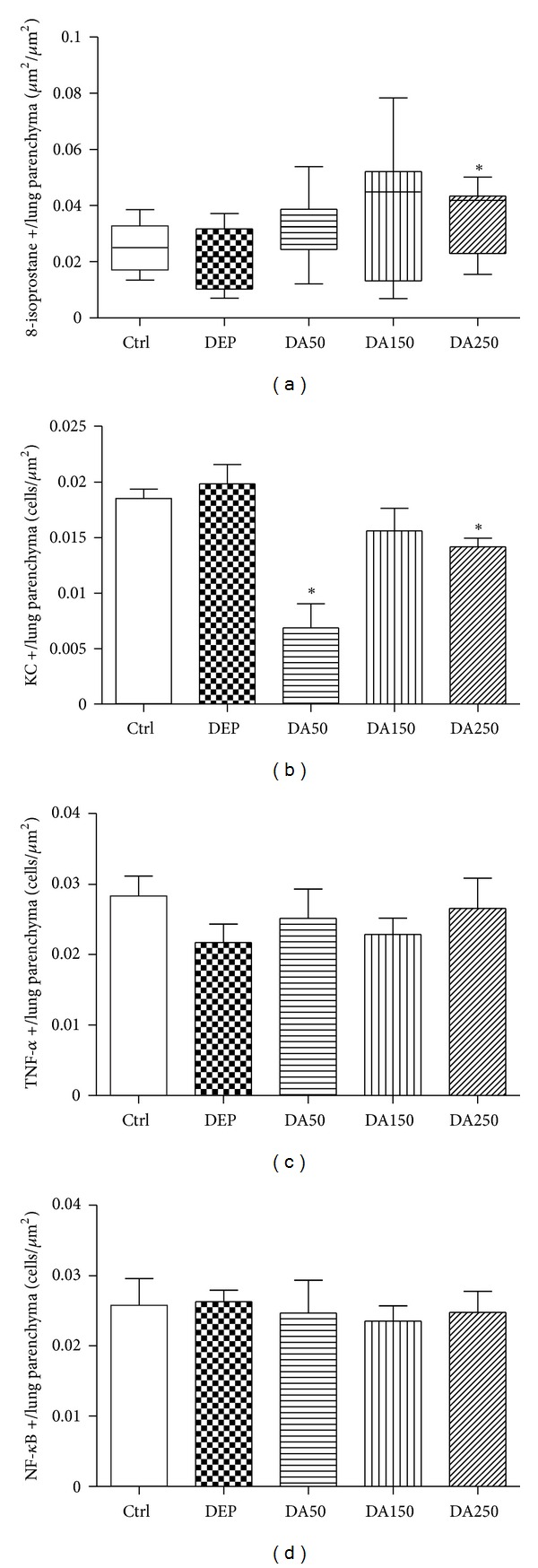
8-isoprostane (a), KC (b), TNF-*α* (c) and NF-*κ*B (d) in the alveolar parenchyma. Values represent means ± SEM or medians (interquartile range). Ctrl: control; animals received an intranasal instillation of 10 *μ*L saline solution and were treated orally with 100 *μ*L of cashew nut oil (CNO). DEP: intranasal instillation of 50 *μ*g DEP/10 *μ*L of saline and treated orally with 100 *μ*L of CNO. DA50: intranasal instillation of 50 *μ*g DEP/10 *μ*L of saline and treated orally with 50 mg/kg of AAs. DA150: intranasal instillation of 50 *μ*g DEP/10 *μ*L of saline and treated orally with 150 mg/kg of AAs. DA250: intranasal instillation of 50 *μ*g DEP/10 *μ*L of saline and treated orally with 250 mg/kg of AAs. KC: keratinocyte chemoattractant. TNF-*α*: tumor necrosis factor-alpha. NF-*κ*B: nuclear factor-kappa B. ^#^ *P* < 0.001 relative to Ctrl.  *P < 0.05 relative to DEP.

**Figure 8:**
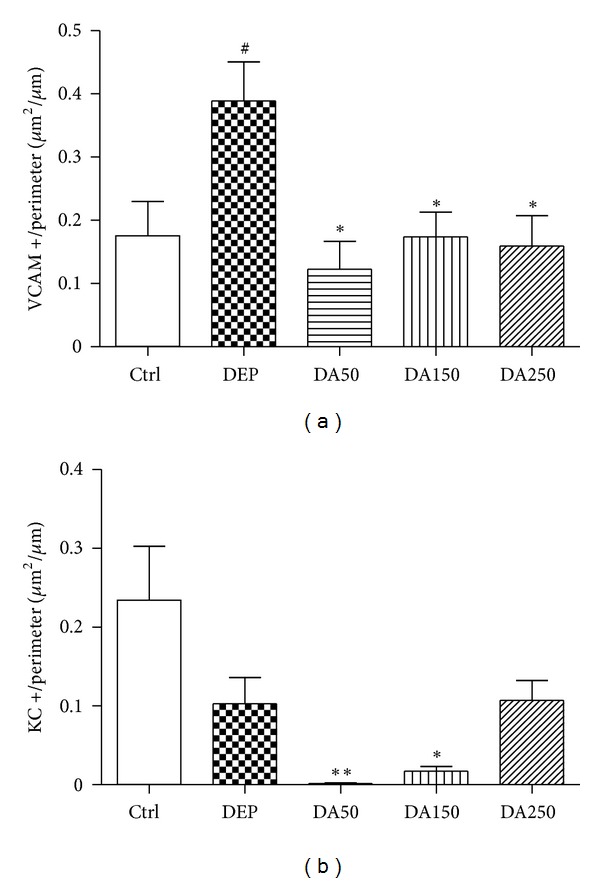
Vascular adhesion molecule (VCAM) (a) and KC (b) positive areas in peribronchiolar vessels. Values represent means ± SEM. Ctrl: control; animals received an intranasal instillation of 10 *μ*L saline solution and were treated orally with 100 *μ*L of cashew nut oil (CNO). DEP: intranasal instillation of 50 *μ*g DEP/10 *μ*L of saline and treated orally with 100 *μ*L of CNO. DA50: intranasal instillation of 50 *μ*g DEP/10 *μ*L of saline and treated orally with 50 mg/kg of AAs. DA150: intranasal instillation of 50 *μ*g DEP/10 *μ*L of saline and treated orally with 150 mg/kg of AAs. DA250: intranasal instillation of 50 *μ*g DEP/10 *μ*L of saline and treated orally with 250 mg/kg of AAs. KC: keratinocyte chemoattractant. ^#^ *P* < 0.05 relative to Ctrl. *P < 0.05 relative to DEP. **P < 0.001 relative to DEP.

**Figure 9:**
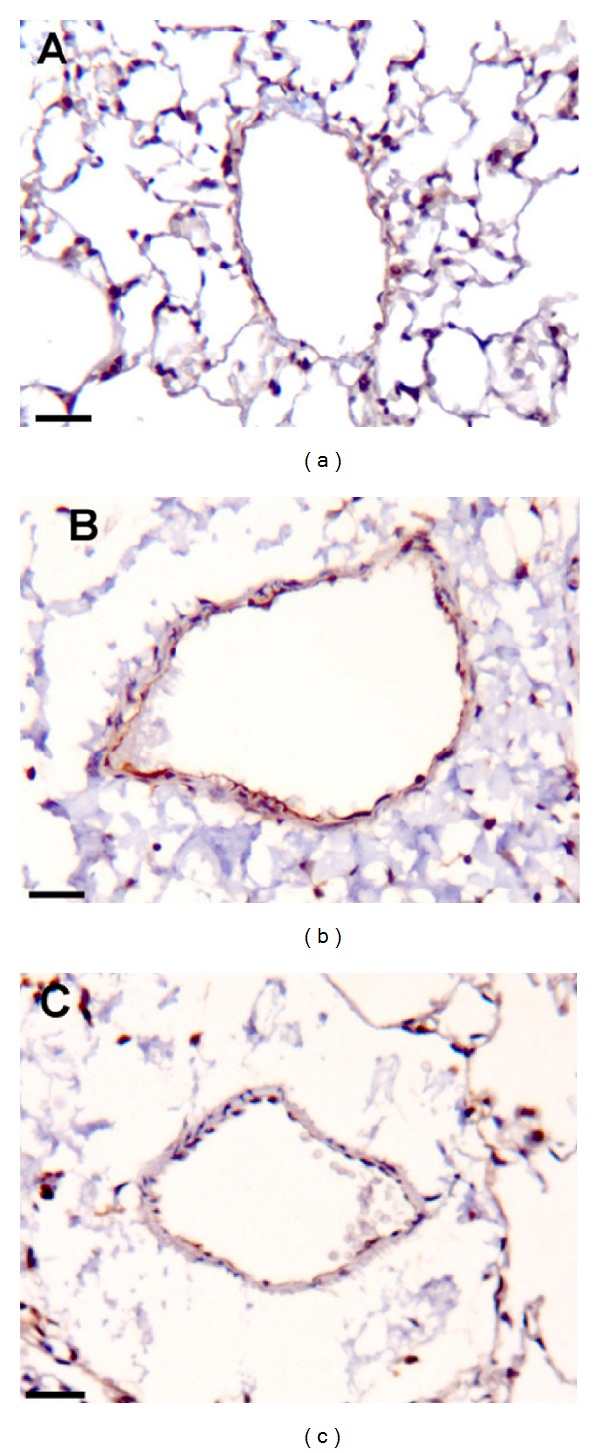
Photomicrographs depicting the increased vascular adhesion molecule (VCAM) area in peribronchiolar vessels of lung tissue. In the DEP-treated group (b) compared to both the Ctrl group (a) and to the group that received 50 mg/kg of anacardic acids (c) (Immunohistochemistry). Scale bars = 25 *μ*m.

The panoramic view of the inflammatory response caused by 50 *μ*g of DEP in the lung tissue compared to Ctrl group was demonstrated in Figure 4. Compared with the Ctrl group, the DEP-treated group demonstrated a 3-fold increase in the influx of neutrophils (*P* < 0.001; Figure 5); however, a decrease in the density of neutrophils was observed in the DA50 and DA150 groups relative to the DEP-treated group (*P* < 0.001, Figure 6(a)). A statistically significant difference was not detected for the density of cells expressing macrophages (Figure 6(b)) in alveolar parenchyma among the DEP and Ctrl groups. Additionally, a decrease in macrophage density was observed in the DA50 and DA150 groups, compared with the DEP group (*P* < 0.05). A significant difference was not detected for the 8-isoprostane-positive area between the DEP and Ctrl groups (Figure 7(a)), although the group that received 250 mg/kg of AAs demonstrated increased values, compared with those for the DEP group (*P* < 0.05). A significant difference was not detected for cells immunostained for KC (Figure 7(b)) in the DEP group, compared with the Ctrl group. DA50 and DA250 groups demonstrated a decreased KC-positive cells, compared with the DEP group (*P* < 0.001 and *P* < 0.05, resp.). There was no significant difference for cells staining positively for the expression of TNF-*α* (Figure 7(c)) and NF-*κ*B (Figure 7(d)) in the lung tissue among the groups. In peribronchiolar vessels (Figure 8(a)), DEP exposure resulted in increased VCAM expression (2-fold), compared with that in the Ctrl group (*P* < 0.05). All animals that received doses of AAs demonstrated decreased expression, compared with those in the DEP-treated animals (*P* < 0.05) as depicted for the DA50 group (Figure 9). A significant difference was not detected for areas positively immunostained for KC (Figure 8(b)) in peribronchiolar vessels in the DEP-treated group, compared with the Ctrl group; however, decreased expression of KC was observed in DA50- and DA150-treated mice, compared with the DEP-treated mice (*P* < 0.001 and *P* < 0.05, resp.).

## 4. Discussion

In the present study, we demonstrated that AAs induce antioxidant and anti-inflammatory responses in a mouse model of DEP-induced lung tissue damage. Thirty days of oral supplementation with 50, 150, or 250 mg/kg of AAs prevented the decrease in GR, GPx, GST, and CAT activities and decreased the expression of VCAM in this model of subacute DEP-induced lung inflammation. At the systemic level (peripheral blood), however, the same protective effects on antioxidant enzymes were not observed.

Animals that received a 50 mg/kg dose of AAs demonstrated decreased levels of neutrophils and TNF-*α* in the lung parenchyma and in the BALF supernatant, respectively. In this model of subacute, low DEP exposure, the lowest dose of AAs (50 mg/kg) appears to be the most effective. To our knowledge, this is the first study to demonstrate the antioxidant and anti-inflammatory properties of AAs in lung tissue* in vivo*.

We consider our exposure model relevant, because it reflects the urban scenario of a megacity, such as São Paulo. Although the World Health Organization recommends 20 *μ*g/m^3^of particulate matter less than 10 *μ*m (PM_10_), the mean annual concentration of PM_10_ in São Paulo is approximately 40 *μ*g/m^3^. During the winter, the levels of 100 *μ*g/m^3^ or above are frequently observed in this city [7, 46]. 

Results from this murine model of increased oxidative burden caused by DEP and prevented by AAs supplementation provide important information about the GSH-enzymes redox cycle. Oral supplementation with AAs from CNSL (50, 150, and 250 mg/kg) prevented the decrease in the antioxidant enzymes GR, GPx, GST, and CAT caused by DEP exposure. 

The GSH redox system is the most important antioxidant defense system in lung cells [47]. GSH-associated enzymes present in the lower respiratory tract act as a first line of defense against sustained oxidative challenges [48, 49]. This system uses GSH as a substrate for GPx and GST in the detoxification of peroxides, including H_2_O_2_, lipid peroxides, and xenobiotic metabolites (such as the ones present in the composition of particulates) [50, 51]. Catalase is an important enzyme responsible for the specific detoxification of H_2_O_2_. 

All of these antioxidant enzymes exhibit decreased activity in the lung tissue following DEP damage but were positively influenced by supplementation with AAs. The same protective effects, however, were not observed in the peripheral blood; only GR activity remained increased in the DEP group compared to the Ctrl group. Several hypotheses may be used to justify this observed discrepancy between lung tissue and systemic responses: (1) DEP exposure only induced local effects in the lung tissue; (2) after 24 h, at sacrifice, the activities of GPx, GST, and CAT returned to basal levels; it was not possible, however, to stabilize the activity of GR, which remained elevated in the DEP group, compared with the other treatment groups, suggesting that this enzyme, yet, acted in the reposition of GSH levels; (3) the partial pressure of oxygen in the arterial blood (PaO_2_) in the lung is higher than PaO_2_ levels in the systemic circulation, predisposing redox events more frequently in the lungs or (4) increased activity of GR in the peripheral blood of DEP-treated mice, compared with that in the other groups, may reflect an initial systemic response against the oxidative challenge. 

AAs are capable of suppressing a variety of prooxidant enzymes involved in the production of ROS [25, 28] and can act directly as divalent metal ion chelators [52, 53], preventing the generation of superoxide anion [19, 52, 54]. Using the xanthine oxidase assay, Kubo et al. [52] demonstrated that at a concentration of 30 *μ*L/mL AAs inhibit the formation of approximately 82% of superoxide anion. The mechanisms for this well-established effect remain unclear, but the degree of unsaturation of the C_15_ alkyl side chain attached to benzene ring is closely related to the observed effects induced by AAs on cell structure and enzymes activities [52, 55, 56]. Taken together, AAs may contribute to the improvement of the oxidative status of compromised lung tissue by facilitating the recovery of GSH and other antioxidant levels. 

The effects of AAs described in this study are attributed to the mixture of AAs isoforms (anacardic acid-1, -2, -3, and -4) which were also used in the majority of *in vivo* studies [23, 32, 52, 54]. Trevisan et al. [19] have previously described that anacardic acid-1 was the more potent antioxidant in the xanthine oxidase assay when compared to anacardic acid-2 and anacardic acid-3. The antioxidant capacity of anacardic acid-1 is explained by the three double bonds in the alkyl side chain, conferring greater antioxidant and enzyme inhibition capacity than the others acids that contain one-two double bonds in their molecules. Therefore, we speculate that the major antioxidant effects observed in this study may probably be assigned to anacardic acid-1.

In future studies, it will be interesting to analyze *in vivo* AA isotypes separately, since recent *in vitro* studies have shown different immunological properties on AAs compounds [57]. In our study, all administered doses of AAs were associated with increased antioxidant enzyme activity whereas only the 50 mg/kg dose of AAs reduced the increase in cytokine levels caused by DEP exposure. In fact at the 150 and 250 mg/kg doses, a significant increase in the concentration of IL-1*β* was observed. A possible reason for this is that the overall exposure to anacardic acid-4 is increased above a threshold in the highest concentrations, whereby it could enhance the production of cytokines as described by Suo et al. [57] for IL-2, interferon-*γ*, IL-4, and IL-5.

A variety of studies have recognized AAs as potential therapeutic substances [23, 30, 52, 56, 58]. Morais et al. [23] described gastroprotective effects of AAs in a dose-related manner. Animals that received 10, 30, or 100 mg/kg of AAs orally have markedly reduced gastric damage induced by ethanol. A dose of 30 mg/kg of AAs was able to inhibit the depletion of GSH, catalase, superoxide dismutase, and nitrate/nitrite, reinforcing the antioxidant potential of anacardic acids.

Currently, there are no reports describing the effects of AAs on the modulation of lung inflammation. Sung et al. [30] demonstrated that AAs inhibit TNF-*α*-activated NF-*κ*B in human lung adenocarcinoma H1299 cells, revealing the potential chemotherapeutic effect of AAs for lung cancer. In our study, AAs did not affect the expression of TNF-*α* and NF-*κ*B in cells in the lung parenchyma. It is possible that this model of subacute, low-DEP exposure was not appropriate for studying such mechanisms. Romieu et al. [2] proposed a hierarchical oxidative stress model that explains the dose-dependent response to air pollutant exposure. This model explains that low levels of exposure lead to the formation of ROS, activating an antioxidant response. At higher levels of exposure, NF-*κ*B and AP-1 transcription is activated, increasing the expression of proinflammatory cytokines.

It is important to note the limitations of the present study. We have not determined the routes of absorption and the metabolism of AAs in mice, and this information is not available for humans. Kubo et al. [52] described several possibilities for these processes: AAs can be absorbed through the intestinal tract and delivered to regions where antioxidants are needed, absorbed as inactive forms, or excreted and not absorbed in human systems. Additionally, we did not study all the possible mechanisms by which AAs can influence the response of lung tissue against DEP. 

## 5. Conclusions

In summary, the antioxidant and anti-inflammatory effects of AAs described in this study using a model of DEP-induced lung damage can be associated with the action of AAs in inhibiting the generation of ROS [19, 25, 52], which can directly or indirectly influence the inflammatory response of the lung. 

Brazil is known for its biological megadiversity, with a large potential for the development of new therapeutic agents derived from native plants as the cashew tree. Much research is still needed in this area. In this study, we have demonstrated that AAs derived from cashew may have a potential role as a therapeutic substance in modulating pulmonary responses by improving antioxidant status. Based on this research, further studies are necessary to detail and describe the mechanisms of action for the observed responses induced by AAs in human health and particularly in the pulmonary system.

## Figures and Tables

**Table 1:** Effects of supplementation with anacardic acids on the cellular profile of the bronchoalveolar lavage fluid in mice instilled with diesel exhaust particles.

Differential cells (×10^4^)	Ctrl	DEP	DA50	DA150	DA250
Total Cells	5.88 ± 2.61	6.76 ± 2.05	8.10 ± 1.61	8.74 ± 1.42	7.52 ± 0.99
Eosinophils	0.00 (0.00)	0.00 (0.06)	0.00 (0.02)	0.00 (0.19)	0.00 (0.00)
Neutrophils	0.04 (0.24)	0.12 (0.13)	0.21 (0.36)	0.20 (0.31)	0.23 (0.10)^a^
Lymphocytes	0.63 ± 0.42	0.47 ± 0.71	0.99 ± 0.56	1.33 ± 0.64^a^	1.17 ± 0.80
Macrophages	4.38 ± 2.24	5.36 ± 1.77	5.86 ± 1.06	6.33 ± 1.32	5.37 ± 0.73
Caliciform cells	0.41 ± 0.26	0.47 ± 0.41	0.57 ± 0.36	0.19 ± 0.19	0.24 ± 0.19
Ciliary cells	0.31 ± 0.27	0.26 ± 0.17	0.41 ± 0.20	0.54 ± 0.47	0.44 ± 0.27

Values are expressed as the means ± SD or as medians (interquartile range). ^a^ *P* < 0.05 significant difference compared to DEP. AAs: anacardic acids. Ctrl: control, animals received an intranasal instillation of 10 *μ*L saline solution and were treated orally with 100 *µ*L of cashew nut oil (CNO). DEP: diesel exhaust particles, intranasal instillation of 50 *μ*g DEP/10 μL of saline and treated orally with 100 *µ*L of CNO. DA50: intranasal instillation of 50 *μ*g DEP/10 μL of saline and treated orally with 50 mg/kg of AAs. DA150: intranasal instillation of 50 μg DEP/10 *μ*L of saline and treated orally with 150 mg/kg of AAs. DA250: intranasal instillation of 50 μg DEP/10 *μ*L of saline and treated orally with 250 mg/kg of AAs.

**Table 2:** Effects of supplementation with anacardic acids on cytokine concentration in the bronchoalveolar lavage fluid in mice instilled with diesel exhaust particles.

Cytokines	Ctrl	DEP	DA50	DA150	DA250
IL-1*β* (*ρ*g/mL)	189.40 ± 43.56	228.52 ± 66.25	181.51 ± 66.30	451.45 ± 138.14^b^	459.72 ± 175.81^b^
TNF-*α* (*ρ*g/mL)	19.84 ± 10.87	118.86 ± 54.24^a^	25.83 ± 6.42^b^	112.44 ± 37.73	130.83 ± 64.67
IL-6 (*ρ*g/mL)	61.96 ± 40.73	74.84 ± 42.84	62.58 ± 46.53	118.11 ± 77.08	103.04 ± 51.44
IL-10 (*ρ*g/mL)	1126.36 ± 463.89	1526.35 ± 538.16	1362.49 ± 217.83	1862.99 ± 646.02	1508.36 ± 499.59

Values are expressed as the means ± SD. ^a^ *P* < 0.001 statistical difference compared to Ctrl. ^b^ *P* < 0.05 significant difference compared to DEP. AAs: anacardic acids. Ctrl: control, animals received an intranasal instillation of 10 *μ*L saline solution and were treated orally with 100 *µ*L of cashew nut oil (CNO). DEP: diesel exhaust particles, intranasal instillation of 50 *μ*g DEP/10 *μ*L of saline and treated orally with 100 *µ*L of CNO. DA50: intranasal instillation of 50 *μ*g DEP/10 μL of saline and treated orally with 50 mg/kg of AAs. DA150: intranasal instillation of 50 μg DEP/10 *μ*L of saline and treated orally with 150 mg/kg of AAs. DA250: intranasal instillation of 50 *μ*g DEP/10 *μ*L of saline and treated orally with 250 mg/kg of AAs.
